# Oncological outcomes of sequential laparoscopic gastrectomy after treatment with camrelizumab combined with nab-paclitaxel plus S-1 for gastric cancer with serosal invasion

**DOI:** 10.3389/fimmu.2024.1322152

**Published:** 2024-01-25

**Authors:** Ju-Li Lin, Mi Lin, Guang-Tan Lin, Qing Zhong, Jun Lu, Chao-Hui Zheng, Jian-Wei Xie, Jia-bin Wang, Chang-Ming Huang, Ping Li

**Affiliations:** ^1^ Department of Gastric Surgery, Fujian Medical University Union Hospital, Fuzhou, Fujian, China; ^2^ Department of General Surgery, Fujian Medical University Union Hospital, Fuzhou, Fujian, China; ^3^ Key Laboratory of Ministry of Education of Gastrointestinal Cancer, Fujian Medical University, Fuzhou, Fujian, China

**Keywords:** Gastric cancer with serous invasion, Camrelizumab, Neoadjuvant chemotherapy, 2-years overall survival rate, 2-year recurrence free survival rate

## Abstract

**Objective:**

To explore the oncological outcomes of sequential laparoscopic gastrectomy after treatment with camrelizumab in combination with nab-paclitaxel plus S-1 for the treatment of gastric cancer with serosal invasion.

**Methods:**

This study is a retrospective cohort study and retrospectively analyzed the clinicopathological data of 128 patients with serosal invasion gastric cancer (cT4NxM0) who received nab-paclitaxel + S-1(SAP) or camrelizumab + nab-paclitaxel + S-1 (C-SAP) regimen and underwent laparoscopy assisted gastrectomy in Fujian Union Hospital from March 2019 to December 2020. The patients were divided into SAP group and C-SAP group. The 2-years overall survival rate, 2-year recurrence free survival rate recurrence rate and initial recurrence time were compared between the two groups.

**Results:**

A total of 128 patients were included, including 90 cases in SAP group and 38 cases in C-SAP group. There were no significant differences in age, gender, gastrectomy method, surgical approach, R0 resection, nerve invasion, vascular invasion, total number of harvested lymph nodes, number of positive lymph nodes and major pathologic response (MPR) rate between the two groups (P>0.05). However, the proportion of ypT0, ypN0 and pCR rate in C-SAP group were significantly higher than those in SAP group (P<0.05). The 2-year OS of C-SAP group (80.7%) was higher than that of SAP group (67.8%), and the difference was not statistically significant (P = 0.112); At 2 years after operation, the recurrence rate of C-SAP group (44.3%) was lower than that of SAP group (55.8%) (P = 0.097); Further analysis showed that the average time to recurrence in the C-SAP group was 18.9 months, which was longer than that in SAP group 13.1 months (P = 0.004); The 2-year recurrence free survival rate in C-SAP group was higher than that in SAP group (P=0.076); There was no significant difference in the overall survival time after recurrence between the two groups (P= 0.097).

**Conclusion:**

Camrelizumab combined with neoadjuvant chemotherapy can improve the proportion of ypT0, ypN0 and pCR in patients, while prolonging the initial recurrence time of patients in the C-SAP group, but did not increase the immunotherapy/chemotherapy related side effects and postoperative complications.

## Introduction

Gastric cancer is the fifth most common malignancy (1) and the third leading cause of cancer-related death(s) worldwide. However, neoadjuvant therapy for locally advanced gastric cancer remains controversial. A previous study (2) reported that neoadjuvant therapy (S-1 combined with nab-paclitaxel) was safe and effective for locally advanced gastric cancer. To improve the feasibility of radical surgery for primary gastric lesions, it is important to develop a treatment scheme with a high tumor remission rate and low toxicity. Accumulating evidence supports the use of immune checkpoint inhibitors for advanced gastric cancer. The KEYNOTE-059 (3) and Check-mate 649 trials confirmed that the programmed death (PD)-1 monoclonal antibody has a significant survival benefit and is safe for advanced, recurrent, or metastatic gastric or gastroesophageal junction adenocarcinoma. Based on the results of KEYNOTE-059 and Check-Mate 649, pembrolizumab and nivolumab were approved as third- and first-line treatments for unresectable and advanced metastatic gastric cancer, respectively. However, their application in patients with locally advanced gastric cancer remains rare.

According to the 8th Edition of the American Joint Committee on Cancer TNM Staging System for Gastric Cancer (4), the clinical stage of patients with cT4aN+M0 gastric cancer was stage III and the 5-year survival rate was 25.9%–43.4%; the clinical stage of cT4bN+M0 patients was stage IVA, and the 5-year survival rate after palliative surgery was only 5%–14.1%. In addition, direct surgical treatment of these patients was associated with low safety and a low resection rate. Preoperative neoadjuvant therapy can effectively improve the resection rate and long-term survival.

Recently, significant progress has been made in neoadjuvant chemotherapy for patients with locally advanced resectable gastric cancer, with Li et al. (5) reporting that laparoscopic distal gastrectomy was safe for patients after neoadjuvant chemotherapy. However, the fibrogenic reaction and cytotoxicity induced by chemotherapy lead to loss of the normal tissue plane, which introduces new technical challenges. However, whether the combined application of immunotherapy affects the perioperative period is unclear. Patients with serous gastric cancer invasion fall between those with locally advanced resectable and those with locally advanced unresectable gastric cancers. Currently, the long-term survival of patients with serous invasion of gastric cancer treated with immune + neoadjuvant chemotherapy combined with surgery has not been reported. As such, this study aimed to explore the oncological outcomes of sequential laparoscopic gastrectomy after treatment with camrelizumab in combination with nab-paclitaxel plus S-1 for the treatment of gastric cancer with serosal invasion to provide evidence-based support for the comprehensive treatment of this patient population.

## Methods

### Study design

This study is a retrospective cohort study. This study was conducted in Fujian Union Hospital from March 2019 to December 2020 retrospectively. The clinicopathological data of patients with serosal invasion gastric cancer (cT4NxM0) who received nab-paclitaxel + S-1(SAP) or camrelizumab + nab-paclitaxel + S-1 (C-SAP) regimen and underwent laparoscopy assisted gastrectomy were retrospectively analyzed.

### Participants

Inclusion criteria: 1. gastric adenocarcinoma confirmed by gastroscopy and pathology before operation; 2. the clinical staging of CT and other imaging evaluation included: cT4; 3. lymph nodes N1 to N3; 4. patients with M0 without distant metastasis from liver, lung, peritoneum and other places evaluated by preoperative CT imaging were included; 5. The ECOG score was 0-2, and the blood indexes, liver and kidney function, cardiopulmonary function could tolerate chemotherapy or surgery. Exclusion criteria: 1. incomplete pathological diagnosis data; 2. patients with gastric stump cancer; 3. gastric cancer patients undergoing emergency surgery; 4. combined with other malignant tumors. Finally, 128 patients were included. Flow diagram was described in **Supplementary Figure 1**.

### Outcome measures/end points

The primary end-point was the 2-years overall survival rate, and the secondary end-points included 2-year recurrence free survival rate, initial recurrence time, pCR, MPR and safety.

### Neoadjuvant therapy

We divided the patients into two groups according to the different neoadjuvant drug treatments: the SAP group (nab-paclitaxel + S-1), and C-SAP group (camrelizumab + nab-paclitaxel + S-1). The specific scheme was as follows:

The cycle of Nab-paclitaxel + S-1 chemotherapy consisted of the following: Day 1: Intravenous Nab-paclitaxel 260 mg/m² over 30 min. Dose reductions (220 mg/m², 180 mg/m², or 150 mg/m²) were permitted in patients with severe haematological or non-haematological toxicity. Day 1–14: S-1 at 120 mg/day for surface area ≥ 1.5m^2^, 100 mg/day for surface area between 1.25 and 1.5m^2^, and 80 mg/day for surface area < 1.25 m^2^ were given 2 times daily. The next chemotherapy was repeated on the 22nd day.

The cycle of Camrelizumab consisted of the following: Day 1: Intravenous Camrelizumab 200mg.

### Tumor regression grade (TRG)

Tumor regression grade according to Becker criteria (6, 7) included “Grade 1a” (Complete tumor regression i.e., 0% residual tumor per tumor bed), “Grade 1b” (Subtotal tumor regression i.e., <10% residual tumor per tumor bed) “Grade 2” (Partial tumor regression i.e., 10–50% residual tumor per tumor bed), “Grade 3” (Minimal or no tumor regression i.e., >50% residual tumor per tumor bed). MPR is defined as: TRG1a+TRG1b. Pathological complete response: pCR is defined as no invasive disease within an entirely submitted and evaluated gross lesion and histologically negative nodes based on central review.

### Postoperative pathological staging

TNM staging was performed according to the 8th edition of AJCC staging standard in 2016. Methods of lymph node treatment: after the specimens were isolated, the lymph nodes of each group were collected and subpackaged, fixed with 10% formalin solution, sent for pathological examination, and sorted by experienced pathologists. The depth of tumor invasion was divided into T1 (invasion of lamina propria, muscularis or submucosa), T2 (invasion of muscularis propria), T3 (invasion of subserosa, but not invasion of visceral peritoneum or adjacent organ), T4a (invasion of serosa) and T4b (invasion of adjacent organ). The staging of lymph node metastasis was divided into N0 (no regional lymph node metastasis), N1 (1 or 2 regional lymph node metastasis), N2 (3 to 6 regional lymph node metastasis), N3a (7 to 15 regional lymph node metastasis), and N3b (≥16 regional lymph node metastasis) according to the 8th edition AJCC staging of gastric cancer. Similarly, ypTNM staging was divided into stage I, stage II, stage III and IV according to the 8th Edition AJCC staging of gastric cancer. If the patient is diagnosed as cT4b before suegery and the postoperative pathology indicates ypT3 or below, it is considered as R0 resection.

### Efficacy evaluation of solid tumor

Tumor response was assessed response evaluation criteria in solid tumors (RECIST), version 1.1 (8): target lesion evaluation criteria (1) CR: all (non lymph node) target lesions diSAPpeared, emphasizing that the short diameter of all original pathological lymph nodes (including target lesions and non target lesions) was<10mm after treatment. (2) PR: the total length and diameter of all target lesions decreased by 30% or more. (3) SD: the change is between PR and PD. (4) PD: the total length and diameter of all target lesions increased by at least 20%, and it was emphasized that the absolute value of the total length and diameter increase was more than 5mm; Or new lesions appear. When lymph nodes were evaluated as target lesions, it was judged that the sum of the long diameters of CR target lesions did not include lymph nodes, as long as the short diameters of all lymph nodes were<10mm; When judging PR, SD and PD, the short diameter of lymph nodes was added to the long diameter of all other target lesions for comparison before and after treatment.

### Surgical indications

After neoadjuvant therapy every 2 cycles, all patients need to review abdominal enhanced CT. Fasting for 6-12 hours before the examination. In addition, the patient drank 600-1000 ml of water to expand the stomach before CT examination. Generally, after 4-6 cycles of neoadjuvant therapy, the operation plan is formulated after multidisciplinary consultation according to the tumor regression grade.

The scope of gastric resection was selected according to the Japanese “Regulations on the treatment of gastric cancer” and the lymph node dissection around the stomach was performed. According to the Japanese Gastric Cancer Treatment Guidelines (5th edition) (9), we perform D2 lymph node dissection. For the distal stomach, we perform lymph node dissection for No.1,3,4sb,5,6,7,8a,9,11p,12a. For the entire stomach, we perform lymph node dissection for No.1-7,8a,9,11p,12a. Standard lymphadenectomy sequences and resection methods were performed as described in the Laparoscopic Gastrectomy for Gastric Cancer (10). LN dissection at station 10 was performed as a selective dissection: (1) When the primary tumor is located in the upper or middle part of the stomach and invades the greater curvature, and (2) When preoperative imaging or intraoperative findings show enlarged lymph nodes in the splenic hilum area, a lymph node dissection for the No.10 region is performed. When No. 14v nodes were highly suspicious for tumor involvement, a lymph node dissection for the No.14v region is performed. This is also a selective dissection.

### Surgery related complications and side events of chemotherapy or immunotherapy

The incidence of surgical complications is based on the number of patients who received surgical treatment as the denominator, and the number of patients with any of the following intraoperative/postoperative complications is the numerator ratio. The standard of intraoperative/postoperative complications refers to the early and late surgical complications mentioned in the intraoperative and postoperative observation items. The severity of complications was graded according to Clavien–Dindo (11) complication scoring system. IIIa and above were serious complications, **Supplementary Table 1**.

The adverse Events of chemotherapy and immunotherapy are classified into grade 0-IV according to the Common Terminology Criteria for Adverse Events (CTCAE) version 5.0 (12), **Supplementary Table 2**.

### Postoperative follow-up

The endpoints of this study include OS and RFS, where OS is defined as the time from surgery to death from any cause. The outcomes include tumor related death, non tumor related death, loss of follow-up, and survival. RFS is defined as the time from the beginning of surgery to the recurrence or death of gastric cancer. According to the follow-up strategy of the Japanese gastric cancer treatment guidelines, patients are followed up every 3 months for the first 2 years and every 6 months for the following 3-5 years. The follow-up routine examination items include physical examination, laboratory examination (CA19-9, CEA, CA72-4), chest X-ray, abdominal ultrasound, CT, and annual gastroscopy examination. The follow-up methods include outpatient follow-up, phone calls, letters, and doorstep visits. The last follow-up date is due to January 2023.

### Ethic

The human research involved in this study has obtained approval from the Ethics Committee of ethics committee of Fujian Union Hospital (Ethics registration number: 2020YF013-01). The study was conducted in accordance with local laws, regulations, and institutional requirements. Detailed information about medication was provided to the patients, and treatment informed consent forms were signed and provided before administration.

### Statistics method

All of the data were analyzed by SPSS software (SPSS, Chicago, IL, USA), version 25.0. The chi-square test or Fisher’s exact test was used for comparisons of categorical variables. The independent sample t test or the Mann-Whitney U test was used for comparisons of continuous variables. Apply COX regression analysis to analyze factors that affect overall survival rate. RFS and OS were estimated using the Kaplan Meier method, RFS and OS were compared using a log-rank test. When P<0.05, it indicates that the difference is statistically significant.

## Results

### Participants

A total of 128 patients were included in this study and divided into two groups: S-1 + nab-paclixatel (SAP), n = 90; and camrelizumab-SAP (C-SAP), n = 38. The median follow-up was 22 months (range, 1–39 months). There were no statistical differences in age, sex, Eastern Cooperative Oncology Group (i.e., “ECOG”) score, Borrmann type, tumor location, preoperative adjuvant cycles, or tumor differentiation type between the two groups (P > 0.05) (**Table 1**).

**Table 1:** Demographic data before surgery.

Baseline Variable	C-SAP group(n=38)	SAP group(n=90)	P* value
Gender			0.586
Male	21(55.3)	45(50.0)	
Female	17(44.7)	45(50.0)	
Age			0.586
<65	29(76.3)	66(73.3)	
≥65	9(23.7)	24(26.7)	
ECOG			0.772
0	34(89.5)	82(91.1)	
1	4(10.5)	8(8.9)	
Tumor size			
median(cm)	5.5	5.1	0.550
Borrmann type			0.857
2-3	29(76.3)	70(77.8)	
4	9(23.7)	20(22.2)	
Neoadjuvant cycle			0.639
≤3	13(34.2)	27(30.0)	
≥4	25(65.8)	63(70.0)	
Tumor location			0.241
Upper	20(52.6)	44(48.9)	
Middle	12(31.6)	20(22.2)	
Lower	6(15.8)	26(28.9)	
Differentiation			0.582
Well/moderate	12(3.6)	33(36.7)	
Poor/ undifferentiated	26(68.4)	57(63.3)	

### Pathological response

The proportion of ypT0 patients in the C-SAP group was 21.1%, which was significantly higher than that in the SAP group (5.6%) (P = 0.008). The proportion of ypN0 in the C-SAP group (63.2%) was significantly higher than that in the SAP group (38.9%), and the difference was statistically significant (P = 0.012). The proportion of those with pathological complete response (pCR) in the C-SAP group (18.4%) was statistically greater than that in SAP group (5.6%) (P = 0.03). The proportion of patients with TRG grade 1a+1b in the C-SAP group was 39.5%, which was similar to that in the SAP group (25.6%) (P = 0.115). The proportion of nerve invasion in the C-SAP and SAP groups was 28.9% and 42.2%, respectively (P = 0.158). The proportion of vascular invasion in the C-SAP and SAP groups was 26.3% and 36.7%, respectively (P = 0.241). The mean (± SD) number of harvested lymph nodes in the C-SAP and SAP groups was 41.6 ± 18.2 and 43.3 ± 14.1, respectively (P = 0.58). The number of positive lymph nodes in the C-SAP and SAP groups 2.3 ± 5.1 and 4.2 ± 3.1, respectively (P = 0.13). Finally, the proportion of those achieving partial response (PR) in the SAP and C-SAP groups was 92.2% and 90.9% (P = 0.982) (**Table 2**).

**Table 2:** Difference of response between two groups.

Baseline Variable	C-SAP group(n=38)	SAP group(n=90)	P* value
TRG			0.014
TRG1a	9(23.7)	5(5.6)	
TRG1b	6(15.8)	18(20.0)	
TRG2	7(18.4)	31(34.4)	
TRG3	16(42.1)	36(40.0)	
subgroup analysis			0.115
TRG1a-1b	15(39.5)	23(25.6)	
TRG2-3	23(60.5)	67(74.4)	
ypT stage			0.059
T0	8(21.1)	5(5.6)	
T1	3(7.9)	11(12.2)	
T2	4(10.5)	12(13.3)	
T3	16(42.1)	51(56.7)	
T4a	6(15.8)	11(12.2)	
T4b	1(2.6)	0	
ypN stage			0.146
N0	24(63.2)	35(38.9)	
N1	6(15.8)	21(23.3)	
N2	3(7.9)	13(14.4)	
N3a	3(7.9)	16(17.8)	
N3b	2(5.3)	5(5.6)	
ypTNM stage			0.106
pCR	7(18.4)	4(4.4)	
I	4(10.5)	14(15.6)	
II	12(31.6)	33(36.7)	
III-IV	15(39.6)	39(43.3)	
ypT stage			0.008
T0	8(21.1)	5(5.6)	
T1-T4b	30(78.9)	85(94.4)	
ypN stage			0.012
N0	24(63.2)	35(38.9)	
N1-N3b	14(36.8)	55(61.1)	
pCR rate			0.03
pCR	7(18.4)	4(4.4)	
I-IV stage	31(81.6)	86(95.6)	
Nerve invasion			0.158
No	27(71.1)	52(57.8)	
Yes	11(28.9)	38(42.2)	
Vascular invasion			0.241
No	28(73.7)	57(63.3)	
Yes	10(26.3)	33(36.7)	
Harvested lymph nodes			0.58
median	41.6±18.2	43.3±14.1	
Positive lymph nodes			0.13
median	2.3±5.1	4.2±3.1	
Radiological response			0.982
PR	35(92.1)	83(92.2)	
SD	3(7.9)	7(7.8)	

### Intraoperative and postoperative clinicopathological results

The mean estimated blood loss in the C-SAP and SAP groups was 70.3 ± 120.9 ml and 41.9 ± 21.6 ml, a difference that was statistically significant (P = 0.008). The proportion of total gastrectomy in the C-SAP and SAP groups was 89.5% and 86.7%, respectively, with no statistical difference (P = 0.661). Two (5.3%) patients in the C-SAP group were converted to open laparotomy, with no conversion to open surgery in the SAP group (P = 0.109). In the SAP group, 1 (1.1%) patient underwent left partial hepatectomy. The R0 resection rate in the C-SAP and SAP groups was 97.4% and 98.9%, respectively (P = 0.58). Operative duration in the C-SAP and SAP groups was 201.3 ± 62.6 min and 208.6 ± 63.5 min, respectively (P = 0.867). The mean time to start liquid diet in the C-SAP and SAP groups was 3.5 ± 1.2 and 3.6 ± 1.0 days, respectively, (P=0.867). The mean time to start semifluid in the C-SAP and SAP groups was 5.1 ± 0.9 and 5.3 ± 0.7 days, respectively (P = 0.851). The mean length of postoperative hospital stay in the C-SAP and SAP groups was 9.0 ± 5.0 and 9.2 ± 11.9 days, respectively (P = 0.794) (**Table 3**).

**Table 3:** Clinicopathological results after surgery.

Baseline Variable	C-SAP group(n=38)	SAP group(n=90)	P* value
Type of gastrectomy			0.661
Partial	4(10.5)	12(13.3)	
Total	34(89.5)	78(86.7)	
Surgical approach			0.109
Laparoscopy	36(94.7)	90(100)	
Conversion to open laparotomy	2(5.3)	0	
Combination organ dissection			
Partial Left liver		1(1.1)	
Extent of resection			0.526
R0	37(97.4)	89(98.9)	
R1	1(2.6)	1(1.1)	
Operation time (min)	201.3±62.6	208.6±63.5	0.867
Estimated blood loss (ml)	70.3±120.9	41.9±21.6	0.002
Time to start liquid diet(days)	3.5±1.2	3.6±1.0	0.862
Time to start semifluid diet (days)	5.1±0.9	5.3±0.7	0.851
Postoperative hospital stay (days)	9.0±5.0	9.2±11.9	0.794

### Surgery-related complications and chemotherapy/immunotherapy-related side effects

The proportions of overall postoperative complications were 24.2% and 22.1% in the C-SAP and SAP groups, respectively, with no statistically significant difference (P=0.801). The proportion of grade I-II complications in the C-SAP and SAP groups was 18.2% and 18.9%, respectively (P = 0.923). The proportion of grade III complications in the C-SAP and SAP groups was 6.1% and 3.2%, respectively (P = 0.826). Grades IV and V complications did not occur in either group. There was no statistically significant difference in the incidence of complications such as pneumonia, abdominal infection, postoperative bleeding, and anastomotic leakage between the two groups (P > 0.05) (**Supplementary Table 3**).

The side effects of neoadjuvant therapy were also analyzed. The most common (grade 3, 4) side effects included declines in neutrophil and white blood cell counts, and increased plasma aspartate aminotransferase (AST)/alanine aminotransferase (ALT) levels. The proportion of patients exhibiting a decrease in white blood cell count (grade 3, 4) in the C-SAP and SAP groups was 20.0% and 21.1%, respectively (P = 0.984). The proportion of patients exhibiting a decrease in neutrophil count (grade 3, 4) was 22.9% in the C-SAP group and 23.3% in the SAP group (P = 0.955). The proportion of anemia (grade 3, 4) in the C-SAP and SAP groups was 3.0% and 2.1% respectively P = 1.000). The proportion of thrombocytopenia (grade 3, 4) in the C-SAP and SAP groups was 5.7% and 3.3%, respectively (P = 0.619). The proportion of patients exhibiting an increase in plasma AST/ALT level in the C-SAP group was 25.7% and 13.3% in the SAP group (P = 0.096). The proportion of febrile neutropenia in the C-SAP and SAP groups was 2.9% and 6.7 (P = 0.649) (**Supplementary Table 4**).

### Two-year overall survival rate after surgery

**Figure 1:**
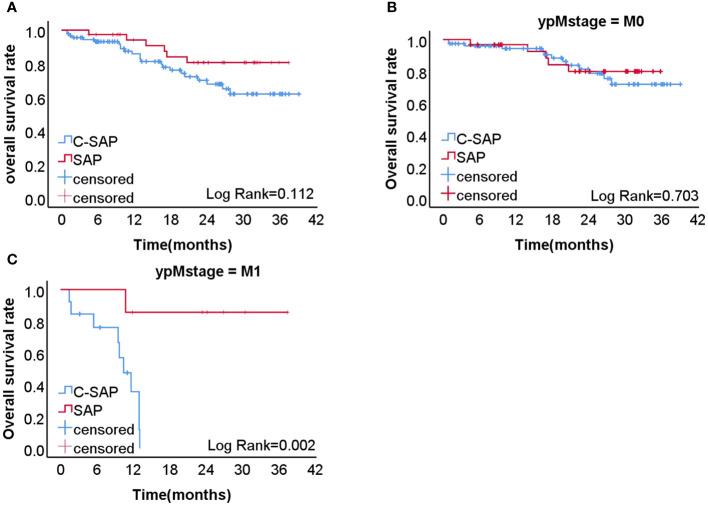
**(A)** the 2-years overall survival rate of C-SAP group (80.7%) was higher than that of SAP group (67.8%) (P=0.112). **(B)** In M0 patients, the 2-years overall survival rate of C-SAP group (79.9%) was similar to that of SAP group (71.9%) (P=0.703). **(C)** In M1 patients, the 2-years overall survival rate of C-SAP group (85.7%) was significantly better than that of SAP group (0%)(P =0.002,).

Analysis revealed that the two-year overall survival (OS) rate in the C-SAP group (80.7%) was higher than that of the SAP group (67.8%) (P = 0.112) (**Figure 1A**). Further stratified analysis revealed that, among M0 patients, the two-year OS rate in the C-SAP group (79.9%) was similar to that of the SAP group (71.9%) (P = 0.703) (**Figure 1B**). Among M1 patients, the two-year OS rate in the C-SAP group (85.7%) was significantly better than that of the SAP group (0%) (P = 0.002) (**Figure 1C**).

The effect of risk factors on the prognosis of the population is illustrated in **Supplementary Figure 1**. The OS rate of ypT0 patients (100.0%) was significantly higher than that of ypT1-T4 patients (68.5%) (P = 0.044) (**Supplementary Figure 2A**). The OS rate of ypN0 patients (74.6%) was higher than that of ypN1-N3b patients (66.2%) (P = 0.21) (**Supplementary Figure 2B**). The OS rate of M0 patients (79.1%) was significantly higher than that of M1 patients (34.9%) (P < 0.001) (**Supplementary Figure 1C**). The OS rate of patients with TRG 1a+1b (76.2%) was higher than that of patients with TRG 2+3 (70.8%) (P = 0.788) (**Supplementary Figure 2D**). The OS rate of patients who underwent ≤ 3 preoperative chemotherapy cycles (75.0%) was higher than that of those who underwent ≥ 4 cycles (70.4%) (P = 0.765) (**Supplementary Figure 2E**). The OS rate of patients who underwent ≤ 3 postoperative chemotherapy cycles (64.0%) was lower than that of those who underwent ≥ 4 cycles (76.2%), a difference that was statistically significant (P = 0.042) (**Supplementary Figure 2F**).

Univariate Cox regression analysis revealed that that ypT0, ≥ 4 postoperative chemotherapy cycles, and M0 were closely associated with patient prognosis. Further multivariate Cox analysis revealed that ≥ 4 postoperative chemotherapy cycles (hazard ratio [HR] 0.418 [95% confidence interval (CI) 0.207–0.891]; P = 0.023) and M1 (HR 5.304 [95% CI 2.464–11.417]; P < 0.001) were risk factors for long-term survival (**Supplementary Table 5**).

### Postoperative recurrence outcomes

**Figure 2:**
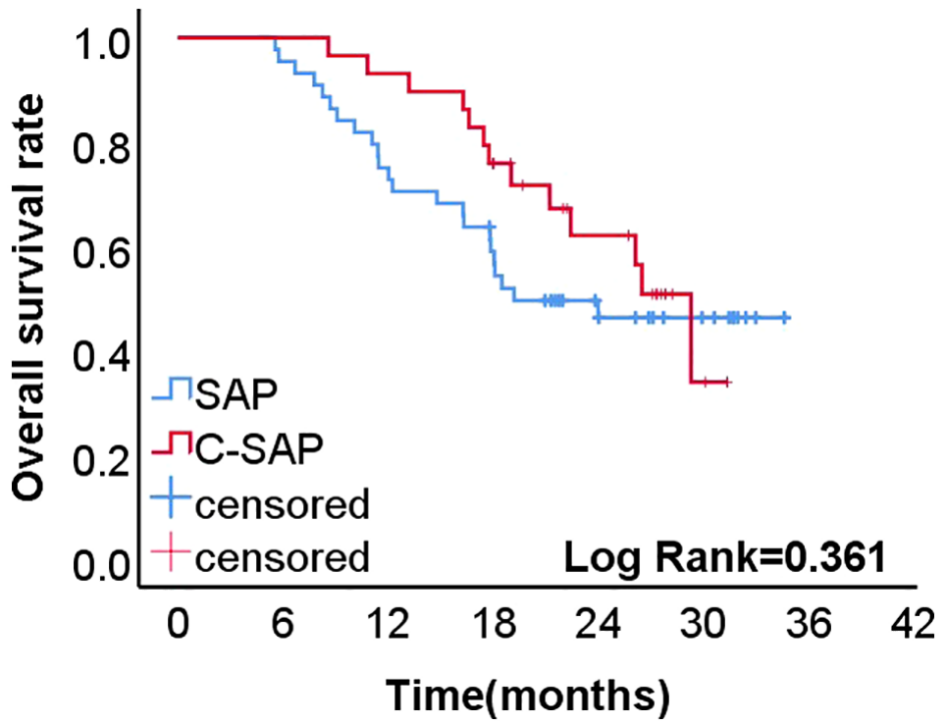
The 2-year recurrence free survival rate of C-SAP group (62.0%) was higher than that of SAP group (46.2%)(P=0.361).

The two-year recurrence free survival (RFS) rate was higher in the C-SAP group (62.0 %) than in the SAP group (46.2%) (P = 0.361) (**Figure 2**).

There was no significant difference in the proportion of recurrence between the C-SAP and SAP groups after 2 years (44.3% versus [vs.] 55.8%, respectively; P = 0.294). However, the average time to recurrence in the C-SAP group was longer (18.9 months) than that in the SAP group (13.1 months) (P = 0.004) (**Table 4**).

**Table 4:** Recurrence within 2 years after surgery.

Baseline Variable	C-SAP group(n=30)	SAP group(n=43)	P* value
Recurrence within 2 years			0.294
Yes	13(43.3)	24(55.8)	
No	17(56.7)	19(44.2)	
Initial recurrence time (month)	18.9	13.1	0.004

The two-year recurrence patterns of the C-SAP and SAP groups are detailed in **Supplementary Table 6**, with distant metastasis being the most common, followed by peritoneal metastasis, and local recurrence being the least common.

There was no statistically significant difference in OS after recurrence between the two groups (P = 0.097) (**Supplementary Figure 3**).

Computed tomography scans for continued immunotherapy after recurrence in the C-SAP group are shown in **Supplementary Figure 4**.

## Discussion

To our knowledge, this is the first study to explore oncological outcomes of sequential laparoscopic gastrectomy after camrelizumab combined with nab-paclitaxel and S-1 (i.e., “C-SAP”) for the treatment of gastric cancer with serosal invasion. The results revealed that camrelizumab combined with neoadjuvant chemotherapy improved the proportion of ypT0, ypN0, and pCR in patients but did not increase immune/chemotherapy-related side effects and postoperative complications. Although it failed to significantly improve the two-year OS and RFS rates, it prolonged the average time to recurrence in patients in the C-SAP group.

Baseline characteristics of the two groups of patients in this study were comparable in terms of general clinical data. The R0 resection rate, operative duration, postoperative recovery, immune/chemotherapy-related side effects, and postoperative complications were not significantly different between the groups.

We compared immune/chemotherapy related side effects in the two groups of patients and found that their incidence was similar; as such, immunotherapy did not result in more side effects. This is similar to the side effects of chemotherapy reported in the ABSOLUTE trial (13) and those for immune/chemotherapy reported in the Neo-PLANET study (14). Therefore, camrelizumab combined with neoadjuvant chemotherapy appears to be safe.

Surgical safety after neoadjuvant therapy is the focus of attention. Li et al. (5) reported that laparoscopic distal gastric cancer surgery appears to have better postoperative safety than open distal gastric cancer surgery in patients with locally advanced gastric cancer receiving neoadjuvant chemotherapy. Li et al. (5) reported that 6 patients (13%) in the laparoscopic surgery group and 20 (40%) in the open surgery group experienced grade II complications. Grade III complications five patients (11%) in the laparoscopic surgery group and two patients (4%) in the open surgery group; Grade IV complications occurred in only 1 patient (2%) in the laparoscopic surgery group, without grade V complications. The proportion of grade II complications in the present study was 18.2% in the C-SAP group and 18.9% in the SAP group, with no statistical difference. The proportion of Grade III complications was 6.1% in the C-SAP group and 3.2% in the SAP group, with no statistical difference. There were no grade IV and grade V complications in either group. Therefore, camrelizumab in combination with neoadjuvant chemotherapy did not increase the incidence of postoperative complications. Intraoperative blood loss was greater in the C-SAP group than in the SAP group. Therefore, more attention should be devoted to intraoperative safety in patients undergoing immunotherapy to avoid intraoperative bleeding.

In the present study, the proportions of those with ypT0, ypN0, and ypCR were higher in the C-SAP group than in the SAP group. The combination of immunotherapy and chemotherapy can effectively alter the overall tumor microenvironment, as well as immune tolerance and immunosuppression, to maintain an effective and persistent antitumor immune response. Increasing evidence supports the use of immunotherapy in combination with chemotherapy for cancer treatment. Chemotherapeutic drugs promote programmed death (PD)-1/PD-ligand1 (PD-L1) expression through multiple signaling pathways (15). Chemotherapy drugs are based on interferon-gamma (IFN- γ) dependent and non-IFN-γ dependent pathways. Depending on the pathway, these drugs can upregulate the expression of PD-L1 by activating different signaling pathways (such as RAS/RAF, PI3K/AKT, JAK/STAT3) and release specific immunosuppressive cytokines, thus weakening the anti-tumor immune response. Therefore, chemotherapy combined with PD-1/PD-L1 inhibitors can enhance antitumor efficacy; as such, the combination of camrelizumab resulted in better tumor response.

The effects of neoadjuvant immunotherapy on several solid tumors have been evaluated. Several phase II studies by the American Society of Clinical Oncology (ASCO) and European Society for Medical Oncology (ESMO) have reported promising pCR rates. Sintilimab combined with the FLOT regimen (fluorouracil + oxaliplatin + docetaxel + leucovorin) (16) and the XELOX regimen (oxaliplatin + capecitabine) (17) for neoadjuvant treatment of gastric cancer resulted in postoperative pCR rates of 18.8% and 23.1%, and major pathologic response (MPR) rates of 62.5% and 53.8%, respectively. The pCR rate for camrelizumab combined with FOLFOX was 8.8% (18). A phase II, single-center, two-arm study (ChiCTR2000030610) enrolled 61 patients who were randomly divided into neoadjuvant camrelizumab + FLOT and neoadjuvant FLOT groups; pCR rates were 11.5% (3/26) and 4.8% (1/21), respectively. Our results revealed that the pCR rate for neoadjuvant chemotherapy combined with camrelizumab was 18.3% and 4.4%, which was higher than that of the neoadjuvant chemotherapy group, with MPR rates of 39.5% and 25.6%, respectively. Recently, a randomized phase II clinical study, Neo-PLANET (NCT03631615) (14) reported the results of the treatment of 36 patients with locally advanced gastric or esophagogastric junction adenocarcinoma with camrelizumab combined with neoadjuvant chemoradiotherapy. Compared with our study, Neo-PLANET reported higher pCR (36.4%) and MPR (48.5%) rates.

In this study, the two-year OS rate of patients in the SAP group treated with two-drug chemotherapy (nab-paclitaxel + S-1) was 67.8%. The two-year OS rate was similar to that of patients receiving the three-drug FLOT regimen (68%) but higher than that of patients receiving the ECF/ECX regimen (59%), which was reported in the phase III clinical study FLOT-4 (19, 20). For patients with gastric cancer invading the serosa, although there is no large randomized controlled trial to further confirm long-term survival with the use of nab-paclitaxel plus S-1, the two-drug regimen is a better choice for Asian populations with poor general conditions.

In this study, the two-year OS and RFS rates were 80.7% and 62.0%, respectively, in patients treated with camrelizumab combined with preoperative chemotherapy (i.e., “C-SAP”). The phase II clinical study Neo-PLANET (14) reported two-year OS and RFS rates of 76.1% and 66.9%, respectively, after sequential gastrectomy after camrelizumab in combination with neoadjuvant chemoradiotherapy, which was similar to the results of our study. Compared with the Neo-PLANET study (14), radiotherapy was not used in the present study; therefore, the related side effects of radiotherapy were reduced. Because the sample sizes in this study and the Neo-PLANET study (14) were small, larger sample sizes are needed to accumulate more evidence. Although there was no statistical difference in the two-year OS and RFS rates between the two groups in this study, the 2-years OS and 2-years RFS of patients in the C-SAP group were higher than those in the SAP group, and the survival curve demonstrated advantages, which appeared to yield long-term survival benefits. We also found that the initial time to recurrence in the C-SAP group was 18.9 months, which was longer than that in the SAP group (13.1 months) (P = 0.004). The two-year recurrence rate was lower in the C-SAP group (44.3%) than in the SAP group (55.8%) (P = 0.076). The combination of camrelizumab appeared to prolong the time to recurrence time and reduce the recurrence rate. Studies, such as KEYNOTE-059 (3) and Check-mate 649, have confirmed the survival benefits of immunotherapy for nonresectable advanced metastatic gastric cancer. Combined with the results of this study and the conclusions of Check-mate 649 and other studies, gastric cancer surgery after a specific number of cycles of immunotherapy combined with preoperative chemotherapy in a specific M1 population may benefit patients. However, more evidence-based studies are needed to support this conclusion.

According to Japanese gastric cancer treatment guidelines (6th edition) (21), it is recommended to consider surgical resection after chemotherapy for oligometastasis (such as 16a2/b1 group omental lymph nodes and solitary liver metastasis). For other stage IV gastric cancer patients, if they have a good response to chemotherapy and the response is sustained, conversion surgery can be considered if R0 resection is achievable. We believe that combination immunotherapy can improve the pathological response rate, enabling some patients to achieve pathological complete response (pCR) or major pathological response (MPR), thereby creating the possibility of cure for oligometastatic gastric cancer patients.

The effectiveness of chemotherapy combined with immunotherapy has been demonstrated in advanced first-line treatment and perioperative period, giving us hope for its breakthrough in the conversion treatment of stage IV gastric cancer. The preliminary exploration was conducted in the CO-STAR study (22) by Chinese researchers, which included 56 cases of unresectable stage IV metastatic gastric cancer patients who received treatment with camrelizumab combined with apatinib and chemotherapy to evaluate the feasibility of surgery. The results showed a high response rate and conversion rate for the camrelizumab combined with apatinib and chemotherapy regimen: ORR 61.7%, R0 resection rate 96.6%. For stage IV gastric cancer, although immunotherapy combined with chemotherapy may have a higher conversion rate and can prolong patient survival, further research is needed to determine its feasibility and safety.

As we all know, dMMR/MSI-H patients are the population that benefits from immunotherapy. According to NCCN Gastric Cancer Guidelines, Version 2. 2022 (23), regardless of the HER-2 status, dMMR/MSI-H population should choose a treatment strategy mainly based on immunotherapy. This includes first-line treatment options, and second-line treatment options recommend immunotherapy, including pembrolizumab, nivolumab plus ipilimumab. Pembrolizumab has indications for MSI-H/dMMR solid tumors. The pooled analysis of KN-059, KN-061, and KN-062 studies targeting gastric cancer found that MSI-H gastric cancer patients can achieve good therapeutic effects regardless of treatment line. In the first-line KN-062 study (24) for gastric cancer, MSI-H patients treated with pembrolizumab showed significant superiority over chemotherapy in terms of ORR (57.7 vs. 36.8%), PFS (11.2 vs. 6.6 months), and 2-year survival rate (71 vs. 26%). Due to the low incidence and lack of large-scale high-level evidence in the field of dMMR/MSI-H gastric cancer, the Level I recommendation is temporarily unavailable.

## Limitations

The present study had several limitations, the first of which were its single-center, retrospective design and inherent selection bias. Second, the follow-up period in this study was < 5 years. Whether it is necessary to screen the population according to PD-L1 expression and MSI status, how to screen the real benefit population, and how to adjust postoperative treatment according to neoadjuvant efficacy are unresolved issues in clinical practice that need to be confirmed by the results of an ongoing RCT.

## Conclusion

This study is the first report long-term survival results for of sequential laparoscopic gastrectomy after camrelizumab in combination with nab-paclitaxel plus S-1 for the treatment of gastric cancer with serosal invasion Camrelizumab combined with neoadjuvant chemotherapy improved the proportion of ypT0, ypN0, and pCR in patients, while prolonging the initial time to recurrence of patients in the C-SAP group, but did not increase immunotherapy/chemotherapy-related side effects and postoperative complications. Although it failed to significantly improve two-year OS and RFS rates after surgery, the survival curve exhibited advantages. This study provides clinical evidence supporting the use of PD-1 inhibitors in the neoadjuvant treatment of gastric cancer.

## Data availability statement

The raw data supporting the conclusions of this article will be made available by the authors, without undue reservation.

## Ethics statement

The studies involving humans were approved by ethics committee of Fujian Union Hospital. The studies were conducted in accordance with the local legislation and institutional requirements. The participants provided their written informed consent to participate in this study.

## Author contributions

J-LL: Conceptualization, Methodology, Writing – original draft, Writing – review & editing. ML: Writing – review & editing. G-TL: Writing – review & editing. QZ: Writing – review & editing. JL: Writing – review & editing. CZ: Writing – review & editing. JX: Writing – review & editing. J-BW: Writing – review & editing. CH: Writing – review & editing. PL: Conceptualization, Writing – review & editing.

## Supplementary material

Supplementary Figure 2
**(A–E)** shows the effect of risk factors on the overall survival rate.

Supplementary Figure 3 No statistically significant difference in the overall survival time after recurrence between the two groups (P=0.097).

Supplementary Figure 4 CT for continued immunotherapy after patient recurrence in C-SAP group.
