# Supplementary figures and images for: Oncological outcomes of sequential laparoscopic gastrectomy after treatment with camrelizumab combined with nab-paclitaxel plus S-1 for gastric cancer with serosal invasion
Source: Front Immunol. 2024 Jan 25;15:1322152. doi: 10.3389/fimmu.2024.1322152 (PMC10850348; doi:10.3389/fimmu.2024.1322152)

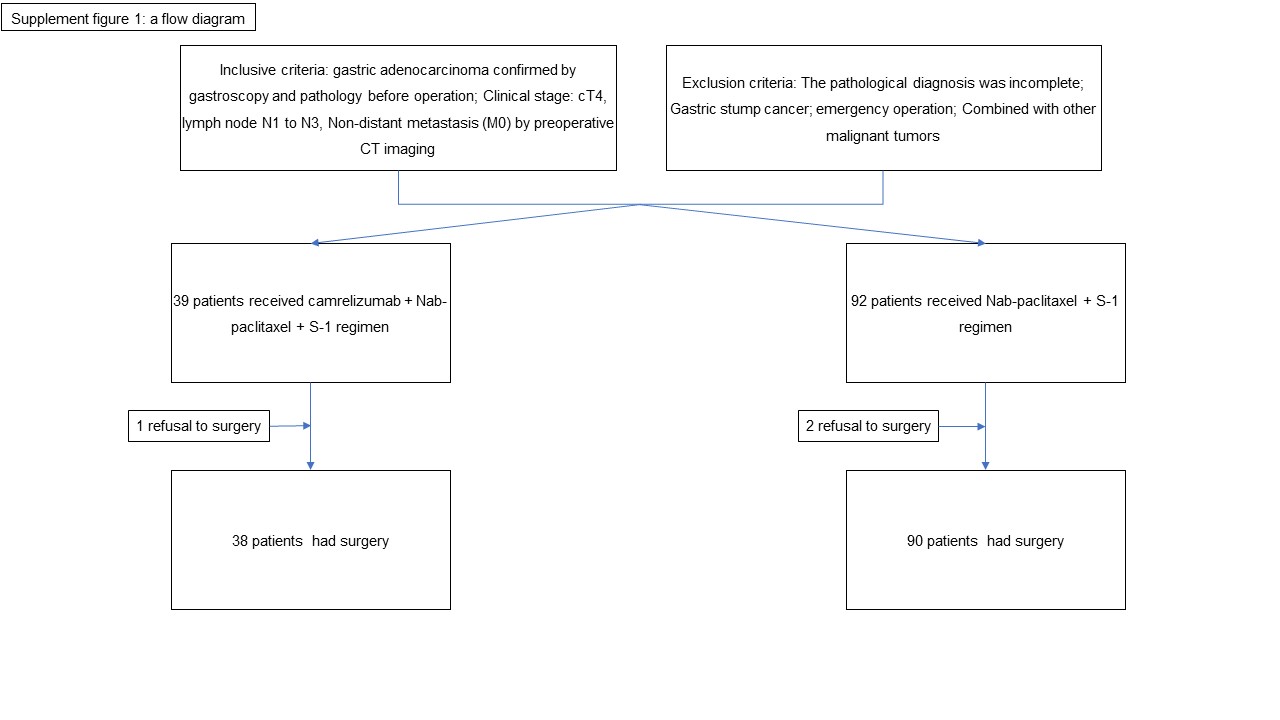

Supplement: Supplementary file 1 [file Image_1.jpeg]

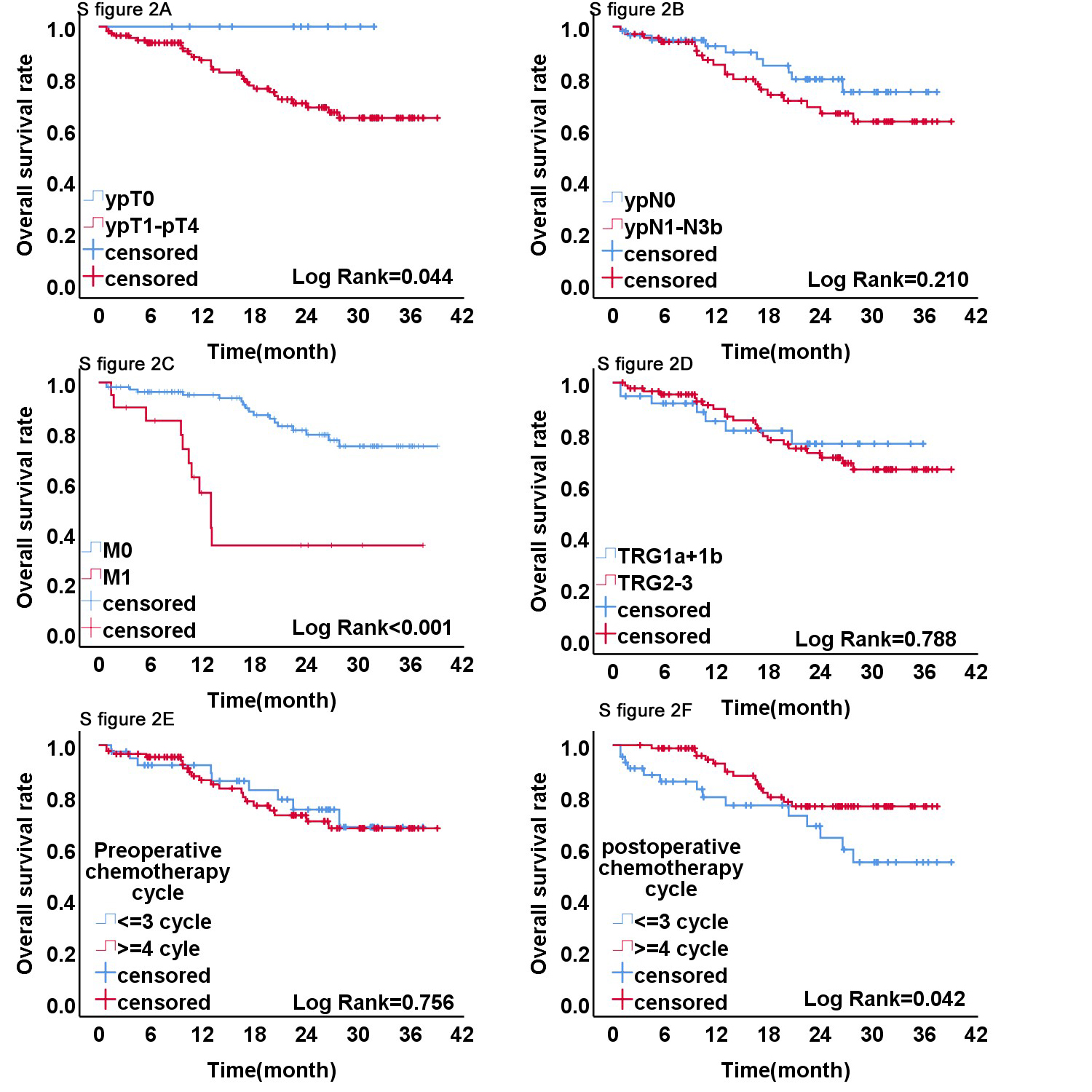

Supplement: Supplementary Figure 2 — (A–E) shows the effect of risk factors on the overall survival rate. [file Image_2.jpeg]

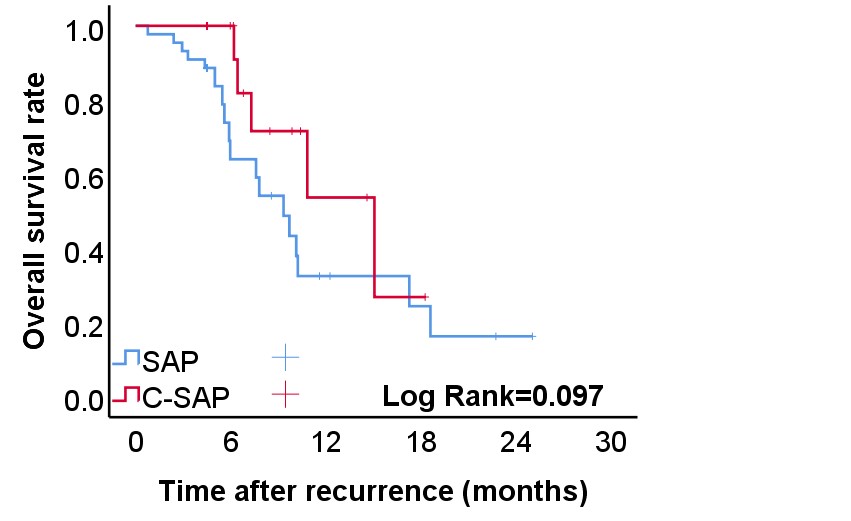

Supplement: Supplementary Figure 3 — No statistically significant difference in the overall survival time after recurrence between the two groups (P=0.097). [file Image_3.jpeg]

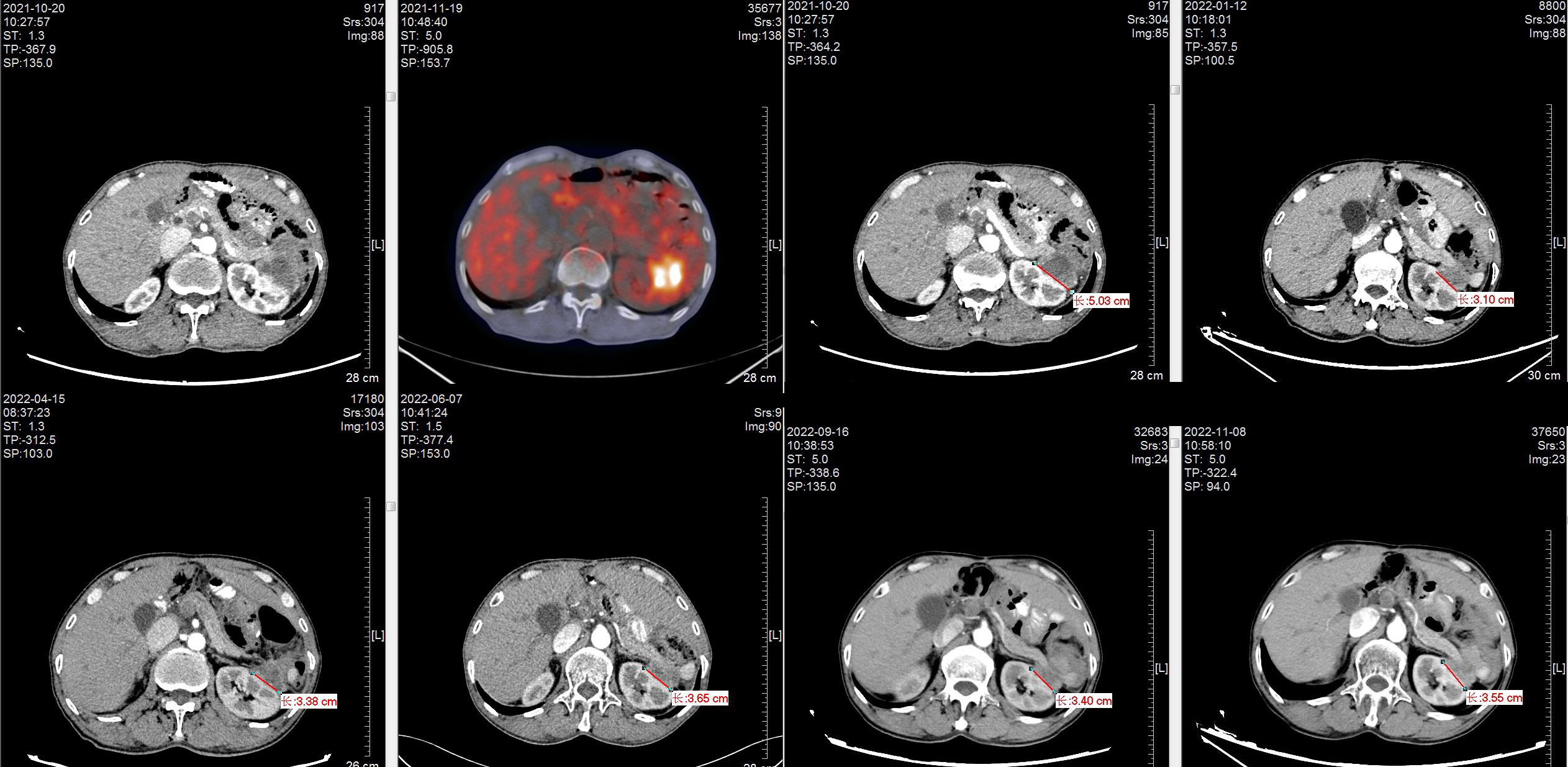

Supplement: Supplementary Figure 4 — CT for continued immunotherapy after patient recurrence in C-SAP group. [file Image_4.jpeg]
