# Supplementary material for: Oncological outcomes of sequential laparoscopic gastrectomy after treatment with camrelizumab combined with nab-paclitaxel plus S-1 for gastric cancer with serosal invasion
Source: Front Immunol. 2024 Jan 25;15:1322152. doi: 10.3389/fimmu.2024.1322152 (PMC10850348; doi:10.3389/fimmu.2024.1322152)
Supplement: Supplementary file 5 [file Table_1.docx]

Supplement table 1 Clavien–Dindo grade

| Grade | Definition |
| --- | --- |
| Grade I | Any deviation from the normal postoperative course without theneed for pharmacological treatment or surgical, endoscopic and radiological interventions.Allowed therapeutic regimens include drugs as antiemetics, antioyretics, analgesics, diuretics and electrolytes and physiotherapy.This grade also includes wound infections opened at the bedside |
| Grade II | Requiring pharmacological treatment with drugs other than suclallowed for grade I complications.Blood transfusions and total parenteral nutrition are also include |
| Grade IIIa | Requiring surgical, endoscopic or radiologicalintervention notnder general anesthesia |
| Grade IIIb | Requiring surgical, endoscopic or radiologicalintervention undgeneral anesthesia |
| Grade IVa | Single organ dysfunction (including dialysis) requiring IC/ICU.nanagement. |
| Grade IVb | Multiorgan dysfunction requiring IC/ICU-management. |
| V | Death of a patient. |

Supplement table 2 Common Terminology Criteria for Adverse Events (CTCAE) version 5.0

| Grade | 0 | I | II | III | IV |
| --- | --- | --- | --- | --- | --- |
| WBC decreased  (10^9^/L) | 4.0 | 3.0-3.9 | 2.0-2.9 | 1.0-1.9 | 1.0 |
| Neutrophil count decreased  (10^9^/L) | 2 | 1.5-1.9 | 1.0-1.4 | 0.5-0.9 | <0.5 |
| Anemia  (g/dL) | >10.0 | 10.0-10.9 | 80.-9.9 | 6.5-7.9 | <6.5 |
| Platelet count decreased  (10^9^/L) | 100-300 | 75-99 | 50-74.9 | 25-49.9 | <25 |
| plasma AST/ALT increase | <1.5*ULN | 1.5~3.0*ULN | 3.0~5.0*ULN | 5.0~20.0*ULN | >20.0*ULN |
| Febrile neutropenia | None |  |  | ANC<1.0*10^9^/L and >38.3°C | Life threatening |

| Supplement table 3 Postoperative complications | | | |
| --- | --- | --- | --- |
| Baseline Variable | C-SAP group(n=33) | SAP group(n=95) | P* value |
| Postoperative  Complication (Yes) | 8(24.2) | 21(22.1) | 0.801 |
| Clavien Dindo grading |  |  |  |
| Grade I-II | 6(18.2) | 18(18.9) | 0.923 |
| Pulmonary infection | 5(15.2) | 14(14.7) | 0.954 |
| Abdominal infection | 1(3.0) | 4(4.2) | 1.000 |
| Grade III | 2(6.1) | 3(3.2) | 0.826 |
| Bleeding | 1(3.0) | 1(1.1) | 1.000 |
| Obstruction | 0 | 0 | NA |
| Anastomotic leakage | 1(3.0) | 2(2.1) | 1 |
| Grade IV | 0 | 0 | NA |
| Grade V | 0 | 0 | NA |

| Supplement table 4 Neoadjuvant treatment adverse effects | | | |
| --- | --- | --- | --- |
| Baseline Variable | C-SAP group(n=35) | SAP group(n=90) | P* value |
| WBC decreased |  |  | 0.984 |
| Grade 0, 1, 2 | 28(80.0) | 71(78.9) |  |
| Grade 3, 4 | 7(20.0) | 19(21.1) |  |
| Neutrophil count decreased |  |  | 0.955 |
| Grade 0, 1, 2 | 27(77.1) | 69(76.7) |  |
| Grade 3, 4 | 8(22.9) | 21(23.3) |  |
| Anemia |  |  | 1.000 |
| Grade 0, 1, 2 | 34(97.1) | 88(97.8) |  |
| Grade 3, 4 | 1(2.9) | 2(2.2) |  |
| Platelet count decreased |  |  | 0.619 |
| Grade 0, 1, 2 | 33(94.3) | 87(96.7) |  |
| Grade 3, 4 | 2(5.7) | 3(3.3) |  |
| Serum AST/ALT increase |  |  | 0.096 |
| Normal | 26(74.3) | 78(86.7) |  |
| Increase | 9(25.7) | 12(13.3) |  |
| Febrile neutropenia |  |  | 0.649 |
| No | 34(97.1) | 56(93.3) |  |
| Yes | 1(2.9) | 4(6.7) |  |

| Supplement table 5 Univariable and multivariable analyses of clinicopathologic variables correlation to overall survival | | | | |
| --- | --- | --- | --- | --- |
|  | Univariable analyses | Multivariable analyses | |  |
| Baseline Variable | P* value | OR | (95% CI) | P* value |
| Gender | 0.704 |  |  |  |
| male |  |  |  |  |
| female |  |  |  |  |
| Age | 0.41 |  |  |  |
| <60 |  |  |  |  |
| ≥60 |  |  |  |  |
| Tumor size | 0.764 |  |  |  |
| ≤5cm |  |  |  |  |
| >5cm |  |  |  |  |
| Borrmann type | 0.64 |  |  |  |
| 2-3 |  |  |  |  |
| 4 |  |  |  |  |
| Tumor location | 0.837 |  |  |  |
| Upper |  |  |  |  |
| Middle |  |  |  |  |
| Lower |  |  |  |  |
| Differentiation | 0.223 |  |  |  |
| well |  |  |  |  |
| poor |  |  |  |  |
| Perioperative Neoadjuvant cycle | 0.765 |  |  |  |
| ≤3 |  |  |  |  |
| ≥4 |  |  |  |  |
| Postoperative adjuvant cycle | 0.042 |  |  |  |
| ≤3 |  | Ref |  |  |
| ≥4 |  | 0.418 | 0.207-0.891 | 0.023 |
| PD-1 treatment | 0.112 |  |  |  |
| No |  |  |  |  |
| Yes |  |  |  |  |
| TRG | 0.778 |  |  |  |
| TRG1a-1b |  |  |  |  |
| TRG2-3 |  |  |  |  |
| ypT stage | 0.044 |  |  |  |
| T0 |  | Ref |  |  |
| T1-T4b |  |  | 0 | 0.975 |
| ypN stage | 0.21 |  |  |  |
| N0 |  |  |  |  |
| N1-N3b |  |  |  |  |
| ypMstage | <0.001 |  |  |  |
| M0 |  | Ref |  |  |
| M1 |  | 5.304 | 2.464-11.417 | <0.001 |

Supplementary table 6 Recurrence model within 2 years after surgery

| Recurrence type within 2 years | C-SAP group(n=13/30) | SAP group(n=24/43) |
| --- | --- | --- |
| Distant metastasis | Retroperitoneal lymph node n=6 | Retroperitoneal lymph node n=7 |
|  | Liver n=2 | Liver n=5 |
|  | Bone n=1 | Bone n=5 |
|  | Mediastinal lymph node n=1 | Mediastinal lymph node n=3 |
|  | Left clavicular lymph node n=3 | Left clavicular lymph node n=2 |
|  | Hilar lymph node n=1 | Paraaortic lymph nodes n=1 |
|  | Lung n=1 | Lung n=1 |
|  | Double accessory ovary n=1 |  |
|  | Navel n=1 | Adrenal gland n=1 |
|  |  | Double accessory ovary n=1 |
| Peritoneal metastasis | Peritoneal metastasis n=2 | Peritoneal metastasis n=7 |
|  |  | Cancerous ascites n=3 |
| Local recurrence | Pancreas n=2 | Pancreas n=1  Anastomotic lymph node n=1 |
|  |  | Duodenum n=1 |
